# Detection and Monitoring of Mining-Induced Seismicity Based on Machine Learning and Template Matching: A Case Study from Dongchuan Copper Mine, China

**DOI:** 10.3390/s24227312

**Published:** 2024-11-15

**Authors:** Tao Wu, Zhikun Liu, Shaopeng Yan

**Affiliations:** 1 Key Laboratory of Intraplate Volcanoes and Earthquakes (China University of Geosciences, Beijing), Ministry of Education, Beijing 100083, China; 2010230039@email.cugb.edu.cn (T.W.); spyan@email.cugb.edu.cn (S.Y.); 2 School of Geophysics and Information Technology, China University of Geosciences, Beijing 100083, China

**Keywords:** mining-induced earthquake, dense seismic array, machine learning-based phase picking, template matching

## Abstract

The detection and monitoring of mining-induced seismicity are essential for understanding the mechanisms behind earthquakes and mitigating seismic hazards. However, traditional underground seismic monitoring networks for mining-induced seismicity are challenging to install and operate, which has limited their widespread application. In recent years, an alternative approach has emerged: utilizing dense seismic arrays at the surface to monitor mining-induced seismicity. This paper proposes a rapid and efficient data processing scheme for the detection and monitoring of mining-induced seismicity based on the surface dense array. The proposed workflow includes machine learning-based phase picking and P-wave first-motion-polarity picking, followed by rapid phase association, precise earthquake location, and template matching for detecting small earthquakes to enhance the completeness of the earthquake catalog. Additionally, it also provides focal mechanism solutions for larger mining-induced events. We applied this workflow to the continuous waveform data from 90 seismic stations over a period of 27 days around the Dongchuan Copper Mine, Yunnan Province, China. Our results yielded 1536 high-quality earthquake locations and two focal mechanism solutions for larger events. By analyzing the spatiotemporal distribution of these events, we are able to investigate the mechanisms of the induced seismic clusters near the Shijiangjun and Lanniping deposits. Our findings highlight the excellent monitoring capability and application potential of the workflow based on machine learning and template matching compared with conventional techniques.

## 1. Introduction

Mining-induced seismicity refers to earthquakes that occur due to the rock rupture caused by the redistribution of subsurface stress resulting from mining excavation [1]. These events are characterized by shallow hypocenters and often exhibit dense clustering, which not only impacts the safe production in mines but also poses threats to the normal lives of surrounding residents. Therefore, effective monitoring of these mining-induced seismicities holds significant importance.

The key to effectively monitoring mining-induced seismicity lies in the establishment of seismic networks. Several decades ago, the countries with significant mining-induced seismic risks, such as South Africa (e.g., [2]) and Poland (e.g., [3]), had already operated underground seismic networks. Although the underground systems can provide near-field recordings of mining-induced seismic events, their installations are challenging and operational costs are high. As a result, most mines have not established independent underground networks for monitoring mining-induced seismicity [4].

In recent years, advancements in artificial intelligence technology have opened up new possibilities for the extensive application of sensors in various fields (e.g., [5]). In this study, we propose a scheme utilizing machine learning-based methods for the detection and monitoring of mining-induced seismicity based on a low-cost dense seismic array at the surface. In the field of seismology, research on earthquake detection (e.g., [6]) and seismic tomography (e.g., [7]) using dense arrays have developed rapidly. However, compared to tectonic earthquakes, mining-induced seismicity exhibits some unique features, such as a higher b-value, more surface wave components [8], and a normal faulting focal mechanism [9]. It is necessary to develop specialized detection and monitoring methods for mining-induced seismicity. To address these challenges, we introduce a machine learning-based method for phase picking from continuous waveforms [10]. Since mining-induced earthquakes typically have small magnitudes, the machine learning-based picking may miss some phases of small events. To overcome this limitation, we adopt a template-matching strategy [6], which selects some larger events as templates to detect earthquakes with smaller magnitudes and lower signal-to-noise ratios through waveform cross-correlation. We also incorporate advanced techniques such as phase association and absolute and relative location methods based on the 3D velocity model. Furthermore, for significant mining-induced events, we employed machine learning methods to determine the P-wave first-motion-polarities, which are then inverted to obtain the focal mechanism solutions.

We apply the aforementioned data processing scheme to the Dongchuan Copper Mine, Yunnan Province, China. The copper mine consists of several large and medium-sized copper deposits such as Sikeshu, Yikeshu, Yinmin, Luoxue, Shijiangjun, Lanniping, and Baixila [11]. Extensive mining activities have led to the formation of large voids, resulting in frequent geological disasters such as roof falls and collapses [12]. According to government bulletins, roof falls and collapses occurred at the Yinmin, Luoxue, and Shijiangjun deposits on (http://www.kmdc.gov.cn/c/2020-11-16/4686961.shtml, accessed on 23 October 2024); (http://www.kmdc.gov.cn/c/2021-01-29/4910453.shtml, accessed on 23 October 2024); (http://www.kmdc.gov.cn/c/2023-02-13/6517175.shtml, accessed on 23 October 2024); and (http://www.kmdc.gov.cn/c/2024-02-23/6812457.shtml, accessed on 23 October 2024). However, due to the lack of underground seismic networks and studies on mining-induced earthquakes in these deposits, there is an urgent need to initiate seismic monitoring for mining-induced events in this region.

To address this need, we utilized continuous waveforms from a temporary dense seismic array deployed around the Dongchuan Copper Mine. We adopted machine learning-based methods for phase picking and first-motion-polarity determination, located the events via seismic phases association and absolute and relative location methods, detected small earthquakes using template matching, and then constructed a high-precision earthquake catalog near the mining area. By combining the analysis of the mining-induced seismicity and focal mechanism solutions for larger earthquakes, we investigated the mechanisms underlying these mining-induced events in this region.

## 2. Data and Methods

### 2.1. Workflow

**Figure 1:**
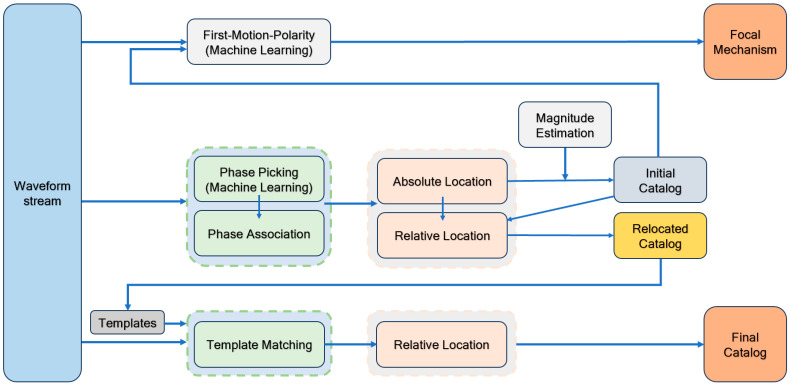
Workflow diagram showing the detection and monitoring of mining-induced earthquakes.

In this work, we develop a comprehensive workflow for the detection and monitoring of mining-induced seismicity. The workflow enables the creation of a rapid initial catalog and focal mechanism solutions for larger events, followed by a more detailed catalog based on template matching and a 3D velocity model. Figure 1 illustrates the diagram of the workflow. Initially, real-time waveform streams undergo routine seismological pre-processing steps, including removing the instrument response, mean, and trend, and band-pass filtering. The removing instrument response is performed to convert the count values of raw seismic records into ground motion displacements. Eliminating the mean and trend helps to remove the baseline drifts and long-term trends caused by factors such as the instrument itself or environmental influences. The waveforms are then band-pass filtered with a frequency band range of 1–30 Hz, which is suitable for mining-induced seismicity and other types of seismicity, such as those associated with hydraulic fracturing and reservoir impoundment. The band-pass filtering could also suppress high-frequency wind noise and cultural noise. Subsequently, a machine learning-based phase picker, PhaseNet [10], is employed to obtain P- and S-wave arrival times. Next, a rapid phase association method, REAL [13], is used to constrain the initial locations of seismic events. An absolute location algorithm, HypoInverse [14], is then applied to produce an initial earthquake catalog, along with calculating the local magnitudes (M_L_) for each event. Based on this initial catalog, two subsequent steps are taken: (a) A relative relocation algorithm, HypoDD [15], based on a 3D velocity model, is conducted, followed by a template matching, Match and Locate [16], to detect the missed events utilizing these events as templates. Finally, the GrowClust3D [17] algorithm, based on waveform cross-correlation differential travel times, is employed to achieve a more complete and accurate final catalog. (b) From the initial catalog, larger earthquakes are selected based on a threshold, and the corresponding P-wave first-motion polarities are determined using a machine learning-based algorithm, DiTingMotion [18]. Then, the focal mechanism solutions are inverted using P-wave first-motion-polarity information. These insights provide crucial data for the analysis of mining-induced earthquakes.

### 2.2. Data

**Figure 2:**
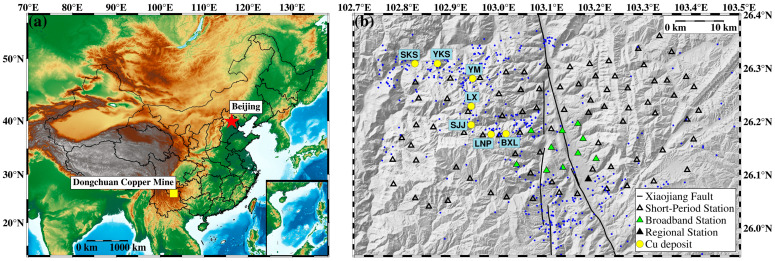
(**a**) Location of Dongchuan Copper Mine in China. (**b**) Distribution of deposits in Dongchuan Copper Mine and seismic stations used in this study. Blue dots indicate the epicenter of the regional network catalog from 2009 to 2021. Abbreviations: SKS, Sikeshu; YKS, Yikeshu; YM, Yinmin; LX, Luoxue; SJJ, Shijiangjun; LNP, Lanniping; BXL, Baixila.

The Dongchuan Copper Mine is situated on the western side of the Xiaojiang fault zone, which serves as the boundary of the Tibetan Plateau (Figure 2a). In early 2021, we deployed a dense seismic array consisting of 80 short-period seismic stations in the middle section of the Xiaojiang fault zone. Each station was equipped with an IGU-16HR 3C seismometer capable of observing frequencies greater than 0.5 Hz. Additionally, we initially set up 10 broadband stations closer to the Xiaojiang fault zone, and each station was equipped with PCS60 seismometers with a frequency band range of 50 Hz-60 s.

These seismic stations formed a dense array with station spacing of approximately 5 km (Figure 2b), and continuous waveforms from 10 January to 5 February 2021 were used in this study. Furthermore, we collected the epicenters of the earthquake catalog from the regional seismic network from 2009–2021, which are displayed in Figure 2b. The figure reveals that the seismicity near the mining area is significantly higher compared to other areas outside the Xiaojiang fault zone. The seismicity is most likely related to the mining activity of the Dongchuan Copper Mine.

### 2.3. Phase Picking Based on Machine Learning

Phase picking is a fundamental task in earthquake detection, and traditional automatic phase-picking methods are usually based on a single feature of waveforms, such as the amplitude or spectrum. For example, the short-term average/long-term average method (STA/LTA) [19] is widely used in earthquake early warning systems due to its simplicity and efficiency. However, its accuracy can be lower when detecting low signal-to-noise ratio events [20]. In recent years, the rapid development of machine learning technology has provided new approaches for earthquake detection. Methods like PhaseNet [10] and EQTransformer [21] have emerged, offering more accurate and efficient solutions. The PhaseNet method employed a U-Net network architecture and used waveforms from the Northern California Earthquake Data Center (NECDC) with 779,514 manually labeled records as its dataset. This dataset was divided into training (623,054 samples), validation (77,866 samples), and test (78,592 samples) datasets. The testing demonstrates that the PhaseNet achieves an F1 score (serviced as a balanced assessment of algorithm performance in both precision and recall) of 0.896 for the P arrivals and 0.801 for the S arrivals when evaluated with a strict threshold for true positive. Through neural network algorithms, it is trained to obtain network parameters that are equivalent to human expert judgment capabilities, allowing it to efficiently pick phases with higher accuracy, particularly for small events with low signal-to-noise ratios.

Compared to EQTransformer, PhaseNet exhibits a higher recall rate (the proportion of true positive cases that are correctly predicted positive [22]). A higher recall rate is particularly important in practical applications for earthquake early warnings and monitoring. Furthermore, false detections can be rejected by phase association algorithms [23]. Therefore, PhaseNet was chosen as the phase picker in our workflow.

**Figure 3:**
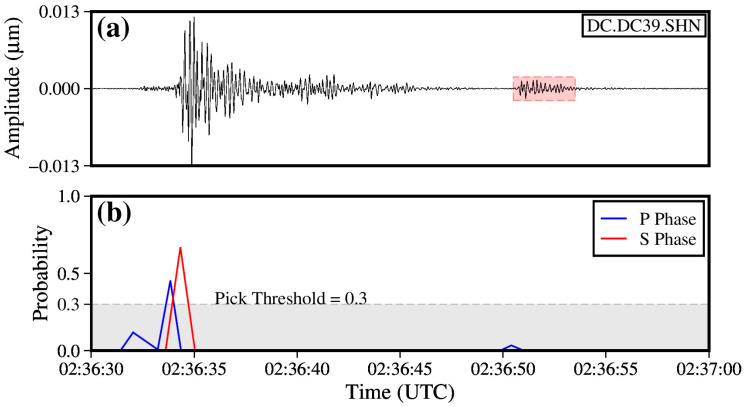
An example of machine learning-based phase picking. (**a**) A segment of 30 s waveforms starting from 02:36:30. (**b**) Probabilities of P-wave phase (blue) and S-wave phase (red). The picking probabilities threshold is set to 0.3 in this study. The event near 20:36:33 can be detected due to its high probability; however, the event within the red rectangle at 02:36:50 cannot be detected.

In this study, we cut continuous three-component waveforms into 30 s segments to predict the probability of the P- and S-wave phases by PhaseNet [10]. We set an empirical picking probabilities threshold of 0.3 for both the P- and S-waves, which generally works well in most local and regional regions [24]. A higher probability threshold (such as 0.5) may lead to higher errors in location due to the decrease in the number of detected phases. As a result, we obtained 311,347 P-wave phases and 317,181 S-wave phases from the waveform data streams. Although machine learning-based phase-picking algorithms can effectively detect some microseismic events, there is still a risk of missing smaller mining-induced events. As shown in Figure 3, the event near 20:36:33 with a magnitude of 0.11 can be detected and located because the probability exceeds the threshold of 0.3. However, the event within the red rectangle at 02:36:50 cannot be detected because of lower picking probability. Nonetheless, the latter event is successfully detected by subsequent template matching, even though its magnitude is only −0.11.

### 2.4. Phase Association

**Figure 4:**
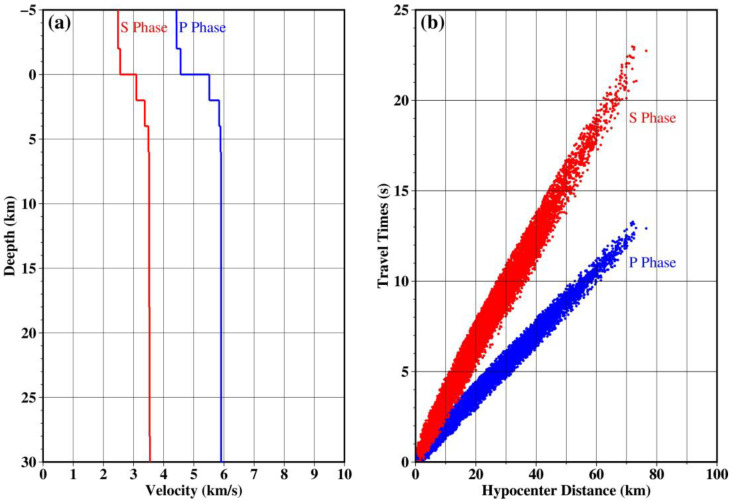
(**a**) The 1D velocity model used for phase association. (**b**) Travel time–hypocentral distance curves of 856 associated earthquakes.

Phase association requires a 1D velocity model, and we established one (Figure 4a) for the study area based on the 3D velocity model, CSES_VM1.0 [25]. We utilized the rapid phase association and location (REAL, [13]) algorithm, which can provide a rough estimate of the earthquake location and origin time using the arrival times of the P- and S-wave phases at each station. The method begins with a 3D grid search around the station with the initiating P-wave pick, followed by the calculation of the P- and S-wave travel times at each station. The grid point with the maximum number of picks and minimum residual is taken as the initial location of the seismic event.

**Figure 5:**
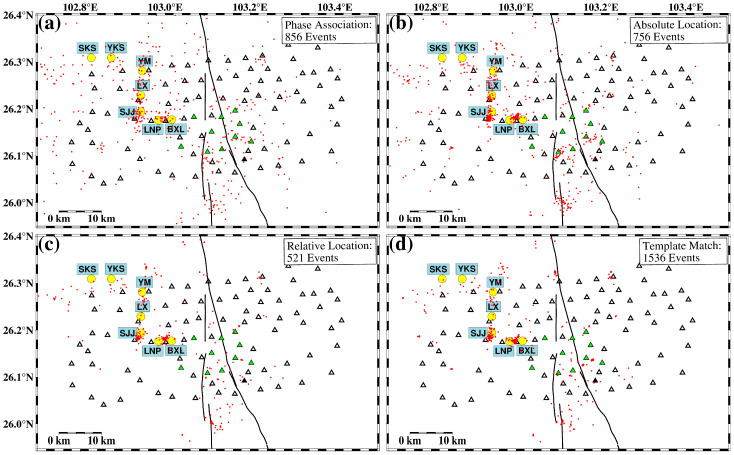
Earthquake catalog comparison between (**a**) phase association, (**b**) absolute location, (**c**) relative location, and (**d**) template matching. Yellow dots indicate the Cu deposits. Red dots indicate the epicenter of seismic events. Open triangles indicate the short-period stations. Green triangles indicate the broadband stations. Black solid triangle indicates the reginal station.

Based on the study area range and station spacing, we established a 3D grid, which has a horizontal range from 0 to 0.14° with an interval of 0.02° and a depth range from 0 to 30 km with an interval of 2 km. To improve the reliability of phase association, we set a threshold requiring at least 5 P-waves and a total of 10 P- and S-wave phases to associate an event. The initial location process yields 856 seismic events (Figure 5a), comprising a total of 15,835 P-wave phases and 14,355 S-wave phases. As can be seen in Figure 4b, due to the accurate 1D velocity model used in this study, the travel time–distance curves of the associated events exhibit a good linear trend without obvious scattered points.

### 2.5. Absolute and Relative Earthquake Location

We applied the HypoInverse algorithm [14], an absolute earthquake location method, to the 856 events listed in the REAL catalog. The output results were then subjected to stringent filtering criteria: both horizontal and depth location errors not exceeding 5 km, travel time residuals not exceeding 0.5 s, and station gap angles not exceeding 300°. A total of 756 high-quality absolute location earthquakes were retained after the filtering, as illustrated in Figure 5b. The absolute location catalog is more concentrated in the mining area and along the fault zone.

To obtain a higher-precision event location, it was necessary to perform relative location after the absolute location. In this study, we used the HypoDD [15] algorithm, which uses the differential travel times for two events that are sufficiently close to each other at the same station for location purposes. The differential travel times can be obtained from the phase picks or measured by cross-correlation of the waveforms.

Since the HypoDD relative location algorithm requires that seismic events be close enough to each other, the event pairing must be performed first. To ensure the quality of the relative location, we set the pairing events with a distance of less than 10 km and both of them must be observed at least at 10 stations simultaneously. This results in a total of 103,995 P-wave and 95,616 S-wave differential travel times. On the basis of this event pairing, the waveform cross-correlation differential travel times are calculated by FDTCC [26,27]. Ultimately, we obtained precise locations for 521 seismic events (Figure 5c). Although the distributions of seismicity in relative and absolute location catalogs are consistent, the results from the relative location exhibit a more obvious pattern of earthquake clusters.

### 2.6. Template Matching

Considering that the machine learning-based phase-picking algorithm used in this study was trained on regional network data, the generalizability of this algorithm for mining-induced seismicity remains to be validated. Moreover, since mining-induced earthquakes are usually small in magnitude, some minor events may be hidden in the background noise and cannot be detected by the aforementioned methods (as seen in the event at 02:36:50 in Figure 3a). Template matching technology has been proven to be effective in detecting weak signals, such as non-volcanic tremors (e.g., [28]), and improving the completeness of earthquake catalogs under strong aftershock activity (e.g., [29]), so we adopted the template-matching method based on waveform cross-correlation technology, Match & Locate [16], in this study.

The waveforms of 521 events from the relative location catalog were used as templates to scan through continuous waveforms. Events with correlation coefficients greater than 0.3 from at least three stations were kept, and their waveform cross-correlation differential travel times were utilized for relocation using the relative earthquake location method [17]. Ultimately, 1536 high-precision seismic events were obtained (Figure 5d).

### 2.7. Magnitude Estimation

To ensure comparability with the regional seismic network earthquake catalog in the study area, we calculated the magnitude of all seismic events. For the relative location catalog, the local magnitude was determined using Chinese national standard (GB 17740—2017) [30]. First, we simulated the waveforms to DD-1-type short-period seismic records [31] and then calculated the magnitudes using the formula

*M*_L_ = log_10_(*A*) + *R*(Δ), (1)

where *A* is the average of the maximum amplitudes in the north–south and east–west components, and *R*(∆) is the regional magnitude calibration function. We calculated the magnitude values from each station for the same event and then obtained the final magnitude by taking their average.

For the events obtained through template matching, we estimated their magnitudes based on the relative amplitude ratio [32] using the following formula

*M*_new_ = *M*_template_ + *c*log_10_(α), (2)

where *M*_template_ is the local magnitude of the template event, α is the amplitude ratio, and *c* is a constant that describes the amplitude–magnitude scaling relationship, which is taken as 1 in this study.

**Figure 6:**
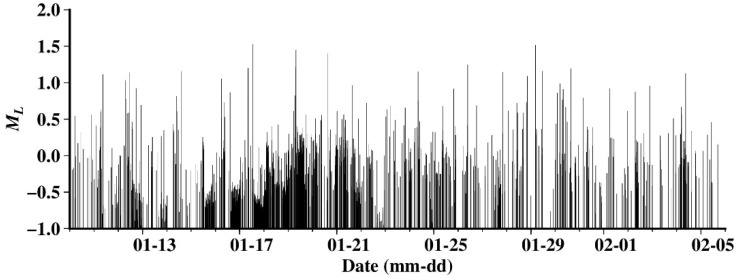
Magnitude–time plot of seismicity during the entire study period.

Figure 6 shows the magnitude–time distribution of all events in this study. It can be seen that during the entire study period, only two earthquakes reached a magnitude of 1.5, while micro-events below 0 magnitude account for a significant proportion.

### 2.8. Machine Learning-Based First-Motion-Polarity Picking and Focal Mechanism Inversion

The focal mechanism solution is crucial for understanding the mechanisms of mining-induced earthquakes. Waveform-based methods for solving focal mechanisms are generally time-consuming due to computationally expensive high-frequency waveform simulations and inaccurate velocity models. Accurate P-wave first-motion-polarity (FMP) can rapidly solve focal mechanisms. Therefore, we introduced a machine learning-based method, the DiTingMotion [18], to determine the first-motion-polarity information.

The DiTingMotion was trained using both DiTing [33] and Southern California Seismic Network (SCSN) first-motion-polarity [34] datasets for accurate first-motion-polarity (“U” and “D”) picking. The DiTing dataset, which has 641,025 high-quality P-wave first-motion-polarity labels, was randomly split into 75%, 10%, and 15% for training, validation, and testing, respectively. The SCSN-FMP contains ~4.84 million seismograms, and ~2.49 M samples from it were selected for training and validation, while ~2.35 M samples were used for testing. The testing demonstrates that the model reached 97.3% (“D”) and 97.86% (“U”) accuracy on the SCSN test dataset and 97.48%(“D”) and 97.77%(“U”) accuracy on the DiTing dataset.

Firstly, for larger magnitude earthquakes, with a threshold of M_L_ 1.0 in this study, we used the initial catalog and associated phase reports to determine the P-wave first-motion-polarities using the machine learning algorithm.

Then, the focal mechanism solutions were inverted as follows. First, the azimuth and take-off angle of the P-wave rays were calculated based on the earthquake location and station locations. Next, according to the computed azimuth and take-off angle, the P-wave first-motion-polarity was marked on the source sphere. Finally, the two nodal planes of the focal mechanism were identified so that the source sphere was divided into four equal-area quadrants with the smallest discrepancy between the observed P-wave first-motion-polarity and those predicted by the focal mechanism model. This results in the inverted focal mechanism solution for the earthquake.

Due to the small magnitude of mining-induced events in this study, only two earthquakes are able to have their focal mechanisms determined.

## 3. Results and Discussion

### 3.1. Comparison with the Regional Seismic Network Catalog

Based on the workflow proposed in this study, we obtained a high-quality location result for 1536 events utilizing 26 days of continuous waveform data (from 10 January to 5 February 2021), recorded by a dense array surrounding the Xiaojiang fault zone (Figure 5d). Notably, although the long-term catalog of the regional network (Figure 2b) indicates active seismicity in the study area, no earthquake events were recorded in the regional network catalog during the study period. This may be due to the low detection capability of the regional seismic network.

**Figure 7:**
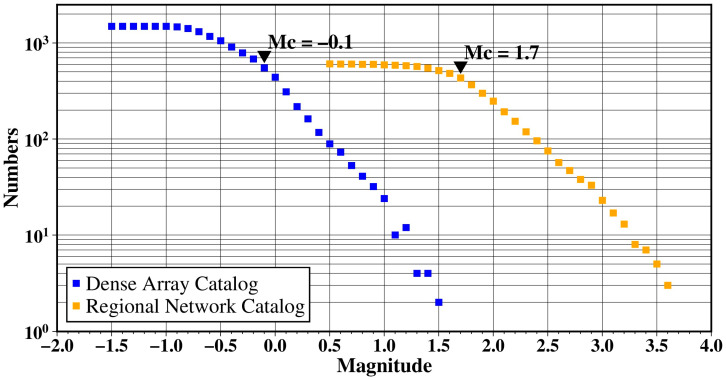
Comparison of magnitude completeness between regional network catalog and dense array catalog obtained in this study.

In seismology, the completeness magnitude (also known as the detection threshold) of an earthquake catalog refers to the minimum magnitude at which all earthquakes are reliably detected and included in the catalog. Figure 7 presents the completeness magnitude for the regional network catalog in the study area between 2008 and 2021, calculated using the maximum curvature method [35], which is approximately 1.7. This is mainly due to the sparse distribution of the regional seismic network, with station spacing typically greater than 50 km. For comparison, we also provide the cumulative magnitude-frequency distribution obtained in this study using dense array data through machine learning-based phase picking and template matching. The resulting completeness magnitude dropped to as low as −0.1. Since the largest earthquake obtained in this study, reaching a magnitude of 1.5, is slightly below the detection capability of the regional seismic network, it was not recorded by the regional network.

Although none of the events detected in this study appeared in the regional network catalog, a comparison between the catalog in this study (Figure 5d) and the regional network catalog (Figure 2b) reveals similar features, which demonstrates the reliability of our detection results. Moreover, we visually inspected the waveforms of most detected microseismic events; it can be confirmed that they are seismic events from waveforms.

**Figure 8:**
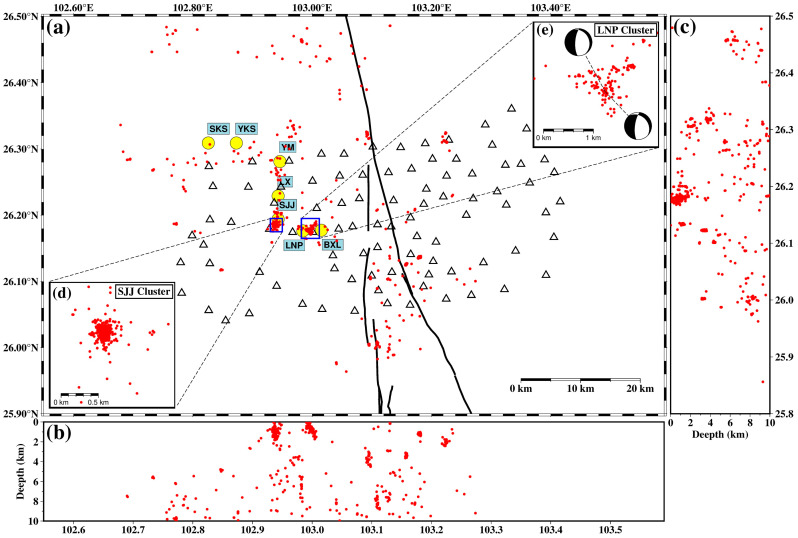
High-precision earthquake catalog (same as Figure 5d) around the Dongchuan Copper Mines using a dense seismic array, machine learning, and template matching. (**a**) Map view. (**b**) West–east cross-section. (**c**) North–south cross-section. (**d**) Enlarged view of SJJ cluster. (**e**) Enlarged view of LNP cluster. Beach balls indicate the focal mechanism. Yellow dots indicate the Cu deposits. Red dots indicate the epicenter of seismic events. Open triangles indicate the seismic stations.

Earthquakes are primarily located near the Xiaojiang fault zone and around various deposits in the Dongchuan Copper Mine. At the western branch of the Xiaojiang fault and the Shijiangjun (SJJ) and Lanniping (LNP) deposits, our earthquake catalog exhibits a more densely distributed pattern, indicating that the use of dense array data significantly improves the precision of earthquake location. Figure 8 presents the horizontal and spatial distributions of the earthquakes obtained in this study. It can be seen that the events are mainly distributed at depths shallower than 10 km. Beneath the Xiaojiang fault zone, there is an approximately vertical distribution of earthquakes at depths of about 4 km and 8 km (Figure 8b), respectively, which is consistent with the high-angle strike-slip of the Xiaojiang fault, possibly reflecting the deep microseismic activity within the fault zone. Another prominent feature is that the microseismic events near the SJJ and LNP deposits are limited to depths shallower than 2 km and are very close to the mine sites, suggesting that it may be related to mining-induced seismicity. Further in-depth study will focus on the two clusters.

### 3.2. Physical Mechanisms of Mining-Induced Seismicity

**Figure 9:**
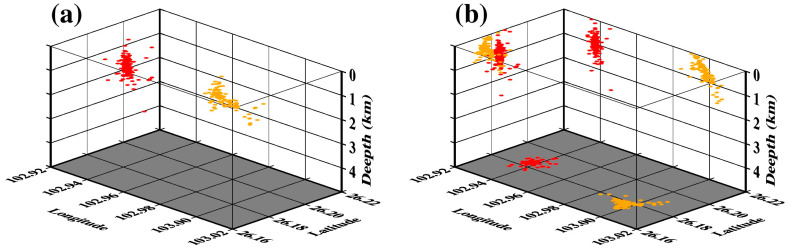
(**a**) The 3D view of the SJJ (red) and LNP (orange) clusters. (**b**) The projections of the SJJ and LNP clusters on each plane in 3D space.

This section focuses on the SJJ and LNP clusters by analyzing their spatial and temporal distributions and focal mechanism solutions to explore the potential physical mechanisms of mining-induced seismicity. Figure 8d and Figure 8e provide enlarged views of the SJJ and LNP clusters, respectively. It can be seen that the distribution of the SJJ cluster is more concentrated, with most events occurring within a circular area of a 0.2 km radius, while the LNP cluster has a more dispersed distribution. Figure 9 better illustrates their spatial distribution. The SJJ cluster is vertically distributed, with the majority of earthquakes occurring at depths between 0.5 km and 1.5 km below the surface. In contrast, the LNP cluster is shallower, mainly located in the range of 0–1 km, and importantly, this cluster tilts towards the northeast direction with an inclination angle of approximately 30° (Figure 9b).

**Figure 10:**
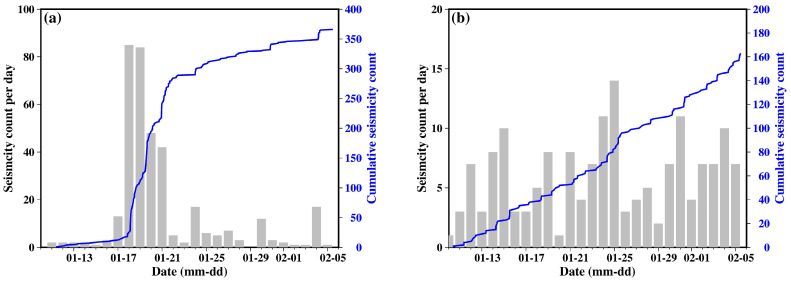
Cumulative number of seismicity and seismicity rate per day for (**a**) SJJ cluster and (**b**) LNP cluster, respectively.

In addition to the differences in spatial distribution, the SJJ and LNP clusters also exhibit distinct temporal evolution patterns. Figure 10 presents the cumulative and daily seismic activity counts for both clusters. The SJJ cluster shows virtually no seismic activity prior to 17 January. However, in the four days following this date, the number of earthquakes rapidly increased from a dozen to nearly 300, with particularly high production on 18–19 January, when more than 80 events occurred each day. In contrast, the growth in the seismic count for the LNP cluster throughout the study period is largely linear, with daily event counts ranging from a few to over a dozen. Even on the day with the highest number of earthquakes (25 January), there are only 14 events recorded.

As previously mentioned, there are no reports of a mining-induced seismicity monitoring network being established in the Dongchuan Copper Mine area. Furthermore, our search through the most comprehensive database of induced earthquakes, HiQuake [36], has not yielded any studies on historically induced seismic events in this region. The differences in temporal and spatial evolutions between the SJJ and LNP clusters suggest distinct physical mechanisms behind them. According to research on the morphology of the LNP orebody [37], its copper deposits are controlled by the NE- and NW-trending faults with dip angles the approximately 38°. This is consistent with the 30° dip angle observed in the LNP cluster. Moreover, both major events in the LNP cluster are of normal faulting type (Figure 8e), which aligns with the nature of the controlling faults. Therefore, we hypothesize that these events may be related to the mining process of the copper deposit. The ongoing excavation of a copper mine could lead to stress changes exceeding certain thresholds, resulting in prolonged mining-induced seismic activity [38].

The SJJ cluster, characterized by a concentration of events over only four days and a vertical distribution, suggests a different mechanism compared to the LNP cluster. The literature reviews have revealed that due to the deep mining operations in the Dongchuan Copper Mine area, extensive shaft construction is common [39]. As previously mentioned, frequent roof falls or collapse accidents occur in this area, typically near the shafts. Thus, we speculate that the SJJ cluster might be associated with roof falls or collapse events at one of the shafts. Additionally, the small magnitude of events in the SJJ cluster, all below 0.5, further suggests that it is more likely related to roof falls or collapse [3].

### 3.3. Implications for Future Induced Seismicity Monitoring

This study has already identified hundreds of mining-induced seismic events in the vicinity of the Dongchuan Copper Mine using less than a month’s worth of observational data. Considering the widespread seismic activity around the mine recorded by regional networks and frequent mining accidents, we hope to strengthen seismic monitoring in this area in the future.

We believe that for the Dongchuan Copper Mine, where there are intensive deposits and multiple mines operating simultaneously, the surface dense seismic array is more practical and effective than establishing an underground monitoring network. Previous studies demonstrated that there was a dense occurrence of small earthquakes before a mining-induced earthquake (Mw 4.1) in Canada [40]. This demonstrates that if the detectable magnitude is small enough, a prediction before potential failure may be possible. Furthermore, fiber-optic sensors have enabled direct observations of interlayer forces [41] and overburden [42] in recent years. By integrating these observations with accurate and rapid monitoring of mining-induced seismic events, along with data on mining progression, there may be significant enhancements in the prediction of mining-induced seismicity. Moreover, making mining operation data (including shaft and tunnel locations, orebody production, and positions and times of blasting) publicly available would significantly enhance the effectiveness of mining-induced seismicity monitoring and contribute to safer production in copper mines.

This study is based on the testing of a software workflow using existing data. If real-time data transmission stations are available [43], this process could also handle real-time data processing and produce rapid preliminary earthquake location results [44]. The refined location results from template-matching and focal mechanism solutions can also be achieved in near-real time. This data processing workflow is not only applicable to mining-induced seismicity monitoring but can also be used for induced earthquakes resulting from shale gas production, reservoir impoundment, and other human activities.

## 4. Conclusions

We present a rapid and effective data processing scheme for the detection and monitoring of mining-induced seismicity based on the surface dense seismic array. The proposed workflow includes automatic phase picking using machine learning-based algorithms, followed by phase association and absolute and relative locations to obtain an initial earthquake catalog. These events are then used as templates to detect smaller earthquakes from continuous waveforms, resulting in a more comprehensive mining-induced earthquake catalog. Additionally, the initial earthquake catalog is utilized to pick P-wave first-motion polarities using machine learning algorithms, which are subsequently employed to invert focal mechanism solutions for larger seismic events. We applied this workflow to the Dongchuan Copper Mine area in Yunnan, China, and obtained 1536 high-quality earthquake locations from 27 days of continuous waveform data recorded by 90 seismic stations, with a completeness magnitude as low as −0.1, significantly lower than the regional network’s threshold of 1.7. Focusing on the temporal and spatial evolution differences between two seismic clusters, LNP and SJJ, we investigated the physical mechanisms behind mining-induced seismicity. We hypothesize that the LNP cluster is triggered by ongoing copper mining activities, which caused changes in rock stress that exceeded a certain threshold, leading to prolonged periods of mining-induced seismicity. In contrast, the SJJ cluster may be associated with roof falls or collapse near a shaft at the mining site.

## Data Availability

The data presented in this study are available upon request from the corresponding author. The data are not publicly available due to privacy concerns.

## References

[1] Foulger G.R., Wilson M.P., Gluyas J.G., Julian B.R., Davies R.J. (2018). Global review of human-induced earthquakes. Earth-Sci. Rev..

[2] Mcgarr A. (1992). Moment tensors of 10 Witwatersrand mine tremors. Pure Appl. Geophys..

[3] Gibowicz S.J., Dmowska R., Saltzman B. (1990). Seismicity induced by mining. Advances in Geophysics.

[4] Li T., Cai M.F., Cai M. (2007). A review of mining-induced seismicity in China. Int. J. Rock. Mech. Min..

[5] Zhang X., Xie X., Tang S., Zhao H., Shi X., Wang L., Wu H., Xiang P. (2024). High-speed railway seismic response prediction using CNN-LSTM hybrid neural network. J. Civ. Struct. Health.

[6] Li Z., Peng Z., Hollis D., Zhu L., McClellan J. (2018). High-resolution seismic event detection using local similarity for Large-N arrays. Sci. Rep..

[7] Mordret A., Roux P., Boué P., Ben-Zion Y. (2019). Shallow three-dimensional structure of the San Jacinto fault zone revealed from ambient noise imaging with a dense seismic array. Geophys. J. Int..

[8] Guo T., Liang S., Zou L. (2021). Using the microseismic template match and locate method to identify microseismic events in the Fushun mining area, Liaoning. Seismol. Geomagn. Obs. Res..

[9] Zhao Y., Liu W., Gao Y. (1995). Distinguishing earthquake, explosion and mine earthquake in Beijing area. Seismol. Geomagn. Obs. Res..

[10] Zhu W., Beroza G.C. (2018). PhaseNet: A deep-neural-network-based seismic arrival time picking method. Geophys. J. Int..

[11] Dai H., Liu J., Zhu W. (2018). Studies on tectonic setting, sedimentary environment and mineralization mechanism of the Yinmin copper deposit in Yunnan Province. Earth Sci. Front..

[12] Du Y., Fang W., Liu Y. (2010). Analyzing of non-pollution type enviromental geology problem of Yinmin mine in Dongchuan cu deposit. J. Earth Sci. Environ..

[13] Zhang M., Ellsworth W.L., Beroza G.C. (2019). Rapid earthquake association and location. Seismol. Res. Lett..

[14] Klein F.W. (2002). User’s guide to HYPOINVERSE-2000, a fortran program to solve for earthquake locations and magnitudes. Open-File Report.

[15] Waldhauser F., Ellsworth W.L. (2000). A double-difference earthquake location algorithm: Method and application to the Northern Hayward Fault, California. Bull. Seismol. Soc. Am..

[16] Zhang M., Wen L. (2015). An effective method for small event detection: Match and locate (M&L). Geophys. J. Int..

[17] Trugman D.T., Chamberlain C.J., Savvaidis A., Lomax A. (2022). GrowClust3D.jl: A julia package for the relative relocation of earthquake hypocenters using 3D velocity models. Seismol. Res. Lett..

[18] Zhao M., Xiao Z., Zhang M., Yang Y., Tang L., Chen S. (2023). DiTingMotion: A deep-learning first-motion-polarity classifier and its application to focal mechanism inversion. Front. Earth Sci..

[19] Stevenson P.R. (1976). Microearthquakes at Flathead Lake, Montana: A study using automatic earthquake processing. Bull. Seismol. Soc. Am..

[20] Chen A., Gao Y. (2019). Developments of research on earthquake detection methods. Prog. Geophys..

[21] Mousavi S.M., Ellsworth W.L., Zhu W., Chuang L.Y., Beroza G.C. (2020). Earthquake transformer-an attentive deep-learning model for simultaneous earthquake detection and phase picking. Nat. Commun..

[22] Powers D.M.W. (2020). Evaluation: From precision, recall and F-measure to ROC, informedness, markedness and correlation. arXiv.

[23] Zhou B., Zhang L., Dai M., Zhao L., Wei F., Zhou Z. (2023). Comparative Study on Seismic Phase Picking of PhaseNet and EQTransformer. J. Geod. Geodyn..

[24] Zhang M., Liu M., Feng T., Wang R., Zhu W. (2022). LOC-FLOW: An End-to-End Machine Learning-Based High-Precision Earthquake Location Workflow. Seismol. Res. Lett..

[25] Wu J., Cai Y., Wang W., Wang W., Wang C., Fang L., Liu Y., Liu J. (2024). Three dimensional velocity model and its tectonic implications at China Seismic Experimental Site, eastern margin of the Tibetan Plateau. Sci. Sin..

[26] Liu M., Li H.Y., Li L., Zhang M., Wang W.T. (2022). Multistage nucleation of the 2021 Yangbi M_S_ 6.4 earthquake, Yunnan, China and its foreshocks. J. Geophys. Res. Solid Earth.

[27] Liu M., Li H.Y., Zhang M., Wang W.T., Yang Y.H., Li L., Chang Z.F., Zhang H.P. (2022). Investigation of the 2013 Eryuan, Yunnan, China Ms 5.5 earthquake sequence: Aftershock migration, seismogenic structure and hazard implication. Tectonophysics.

[28] Shelly D.R., Beroza G.C., Ide S. (2007). Non-volcanic tremor and low-frequency earthquake swarms. Nature.

[29] Peng Z., Zhao P. (2009). Migration of early aftershocks following the 2004 Parkfield earthquake. Nat. Geosci..

[30] (2019). General Ruler for Earthquake Magnitude.

[31] Yang W., Chen G., Meng L., Zang Y., Zhang H., Li J. (2021). Determination of the local magnitudes of small earthquakes using a dense seismic array in the Changning−Zhaotong Shale Gas Field, Southern Sichuan Basin. Earth Planet. Phys..

[32] Shelly D.R., Hardebeck J.L. (2019). Illuminating faulting complexity of the 2017 yellowstone maple creek earthquake swarm. Geophys. Res. Lett..

[33] Zhao M., Xiao Z., Chen S., Fang L. (2023). DiTing: A large-scale Chinese seismic benchmark dataset for artificial intelligence in seismology. Earthq. Sci..

[34] Ross Z.E., Meier M., Hauksson E. (2018). P Wave Arrival Picking and First-Motion Polarity Determination with Deep Learning. J. Geophys. Res. Solid Earth.

[35] Wiemer S., Wyss M. (2000). Minimum magnitude of completeness in earthquake catalogs: Examples from Alaska, the western United States, and Japan. Bull. Seismol. Soc. Am..

[36] Wilson M.P., Foulger G.R., Gluyas J.G., Davies R.J., Julian B.R. (2017). HiQuake: The human-induced earthquake database. Seismol. Res. Lett..

[37] Wang T., Fang W., Du Y. (2012). The morphological characteristics of iron-copper orebody and ore-controlling factors in Lanniping, Dongchuan. Metal. Mine.

[38] Castellanos F., Van der Baan M. (2015). Dynamic triggering of microseismicity in a mine setting. Geophys. J. Int..

[39] Fei Z., Wu D., Yang Z., Tong Z. (2019). Influence analysis of underground mining on the stability of main and auxiliary mineshaft in Dashuigou section of Yinmin copper mine. J. Kunming Metall. Coll..

[40] Kubacki T., Koper K.D., Pankow K.L., McCarter M.K. (2014). Changes in mining-induced seismicity before and after the 2007 Crandall Canyon Mine collapse. J. Geophys. Res. Solid Earth.

[41] Zhang X., Zheng Z., Wang L., Cui H., Xie X., Wu H., Liu X., Gao B., Wang H., Xiang P. (2024). A Quasi-Distributed optic fiber sensing approach for interlayer performance analysis of ballastless Track-Type II plate. Opt. Laser Technol..

[42] Hu T., Hou G.Y., Li Z.X. (2020). The Field Monitoring Experiment of the Roof Strata Movement in Coal Mining Based on DFOS. Sensors.

[43] Li J., Yao H., Wang B., Yang Y., Hu X., Zhang L., Ye B., Yang J., Li X., Liu F. (2022). A real-time AI-assisted seismic monitoring system based on new nodal stations with 4G telemetry and its application in the Yangbi MS 6.4 aftershock monitoring in southwest China. Earthq. Res. Adv..

[44] Zhu W., Hou A.B., Yang R., Datta A., Mousavi S.M., Ellsworth W.L., Beroza G.C. (2023). QuakeFlow: A scalable machine-learning-based earthquake monitoring workflow with cloud computing. Geophys. J. Int..

[45] Wessel P., Luis J.F., Uieda L., Scharroo R., Wobbe F., Smith W.H.F., Tian D. (2019). The Generic Mapping Tools Version 6. Geochem. Geophys. Geosyst..

